# Peripheral nervous system manifestations in a Sandhoff disease mouse model: nerve conduction, myelin structure, lipid analysis

**DOI:** 10.1186/1477-5751-6-8

**Published:** 2007-07-10

**Authors:** Melanie A McNally, Rena C Baek, Robin L Avila, Thomas N Seyfried, Gary R Strichartz, Daniel A Kirschner

**Affiliations:** 1 Biology Department, Boston College, 140 Commonwealth Avenue, Chestnut Hill, MA 02467, USA; 2 Pain Research Center, Department of Anesthesiology, Perioperative and Pain Medicine, Harvard Medical School, Brigham and Women's Hospital, 75 Francis Street, Boston, MA 02115, USA

## Abstract

**Background:**

Sandhoff disease is an inherited lysosomal storage disease caused by a mutation in the gene for the β-subunit (*Hexb *gene) of β-hexosaminidase A (αβ) and B (ββ). The β-subunit together with the GM2 activator protein catabolize ganglioside GM2. This enzyme deficiency results in GM2 accumulation primarily in the central nervous system. To investigate how abnormal GM2 catabolism affects the peripheral nervous system in a mouse model of Sandhoff disease (*Hexb-/-*), we examined the electrophysiology of dissected sciatic nerves, structure of central and peripheral myelin, and lipid composition of the peripheral nervous system.

**Results:**

We detected no significant difference in signal impulse conduction velocity or any consistent change in the frequency-dependent conduction slowing and failure between freshly dissected sciatic nerves from the *Hexb*+/- and *Hexb*-/- mice. The low-angle x-ray diffraction patterns from freshly dissected sciatic and optic nerves of *Hexb*+/- and *Hexb*-/- mice showed normal myelin periods; however, *Hexb*-/- mice displayed a ~10% decrease in the relative amount of compact optic nerve myelin, which is consistent with the previously established reduction in myelin-enriched lipids (cerebrosides and sulfatides) in brains of *Hexb-/- *mice. Finally, analysis of lipid composition revealed that GM2 content was present in the sciatic nerve of the *Hexb*-/- mice (undetectable in *Hexb*+/-).

**Conclusion:**

Our findings demonstrate the absence of significant functional, structural, or compositional abnormalities in the peripheral nervous system of the murine model for Sandhoff disease, but do show the potential value of integrating multiple techniques to evaluate myelin structure and function in nervous system disorders.

## Background

Gangliosides are a diverse class of glycosphingolipids (GSL) involved in cell-to-cell interactions, regulation of cell growth, apoptosis, neuritogenesis, and differentiation of cells [1]. Gangliosidoses, like Tay-Sachs, occur when these lipids are incompletely catabolized due to an inherited enzyme deficiency; GM2 gangliosidoses are characterized by incomplete GM2 catabolism due to the absence of β-hexosaminidase activity. The α- and β-subunits of β-hexosaminidase are encoded by the HEXA and HEXB genes. In non-pathogenic conditions, ganglioside GM2 is degraded to GM3 in the lysosome by the HexA isoenzyme combined with the GM2 activator protein. Without the activity of the HexA isoenzyme, massive lysosomal GM2 accumulation is observed which disrupts the normal cytoarchitecture of the neuronal cells [2]. Sandhoff disease (SD) is an inherited GM2 gangliosidosis that occurs in 1 of every 384,000 live births [3]. Both HexA and HexB are non-functional. Curative therapy for SD and other GSL storage disorders has not yet been elucidated; however, some treatments that have shown promise managing these diseases are enzyme replacement therapy, gene therapy, bone marrow transplant, stem cell therapy, substrate reduction therapy, and caloric restriction [4-8].

The SD mouse model (*Hexb-/-*) shows rapid GM2 accumulation characteristic of early onset SD in patients. By contrast, heterozygotes (*Hexb+/-*) do not display any of these symptoms, express normal ganglioside distribution, and live a normal life span around 2 years [9]. By postnatal day 5, the *Hexb*-/- mice exhibit GM2 and, its asialo derivative, GA2 accumulation in the brain [10]. This accumulation of GM2 parallels neurochemical features of the infantile form of SD. After 3 months, *Hexb*-/- mice begin a steady progression to near complete loss of hind limb movement, excess muscle wasting, especially in the hind limbs, and abnormal motor function. After 4.5 months, *Hexb*-/- mice are unable to move, eat, or drink, and there is a 300% increase of GM2 in the brains of these animals [6]. In the *Hexb*-/- mice, extensive neuronal storage is observed throughout the cerebrum, cerebellum, spinal cord, trigeminal ganglion, retina, and myenteric plexus [9].

Abnormalities in the PNS as part of the pathology of the GM2 gangliosidoses have also been found. Specifically, studies have shown a motor neuron disease phenotype, loss of large diameter myelinated fibers in the peroneal nerve, and abnormal sympathetic nervous skin responses in patients with chronic GM2 gangliosidosis [11-13]. In addition, GM2 accumulation has been detected in anterior horn motor neurons and in the Schwann cells of the dorsal root ganglion in a mouse model of SD [9,14,15]. This mouse model also demonstrates apparent hind-limb paralysis and extensive hypotonia [9]. Despite these studies, SD is commonly considered a disease of the central nervous system (CNS) and elucidation of the peripheral nervous system (PNS) in patients and animal models remains incomplete. To illuminate our understanding of SD as pertaining to the integrity of PNS myelin in the mouse model of SD (*Hexb-/-*), we used electrophysiological methods for function, low-angle x-ray diffraction (XRD) for structure, and high-performance thin-layer chromatography for lipids. Our working hypothesis for the present study was: if the lipid composition of the neuronal or myelin membranes in the PNS was altered due to faulty catabolism of GM2, then changes in the myelin and in nerve electrophysiology would be observed. Classically, XRD is used for periodicity measurements of internodal myelin; here, we also used it to quantitate the relative amount of myelin in whole nerves [16,17]. The results demonstrate the value of integrating multiple techniques to evaluate myelin structure and function and offer a potential strategy that will be useful for future investigations into nervous system disorders that could involve demyelination.

## Results

### Electrophysiological measurements were normal

**Figure 1:**
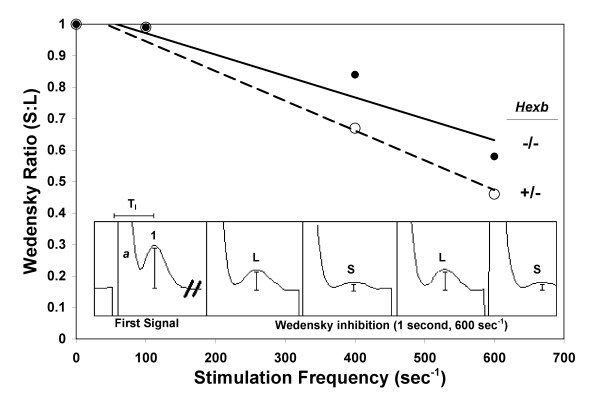
*Wedensky Ratio vs. Stimulation Frequency in Hexb+/- and Hexb-/- Mice*. Wedensky ratios (see Materials and Methods) for *Hexb*+/- (○, dashed line) and *Hexb*-/- (●, solid line) mice were plotted against the stimulation frequency with linear regressions (n = 6–10) to analyze frequency-dependent conduction failure in the two mouse models. As evidenced by the decrease in the Wedensky ratio in both groups, conduction failure after a one second stimulus train increased in alternating CAP signals with increasing stimulation frequency. The slopes of the linear regressions were not different within 95% confidence levels indicating similar conduction failure behavior in the *Hexb*+/- and *Hexb*-/- mice. The first CAP signal recorded during a 1 second supramaximal stimulation at 600 sec^-1 ^is compared to the last four CAP signals in the train (scale conserved). Wedensky inhibition is observed. **T_l_**, latency used for CNCV calculations; **a**, stimulus artifact; **1**, amplitude of first CAP in stimulus train; **L**, **S**, CAP amplitudes after 1 sec of 600 Hz stimulation (1.67 msec between stimuli).

Sciatic nerves from 5 *Hexb+/- *and 7 *Hexb-/- *mice were used for electrophysiological experiments. Compound nerve conduction velocity (CNCV) values of the two groups were not different (Table 1). The sciatic CNCVs of the *Hexb*+/- and *Hexb*-/- mice were 23.6 m/s ± 0.6 and 25.1 m/s ± 0.9, respectively (mean ± SEM). The data show that the CNCV falls significantly more in the *Hexb*-/- mice than the *Hexb*+/- mice when stimulated at 100 sec^-1 ^for 1 second (p < 0.05, two-tailed, unpaired *t*-test). However, this difference was not observed at higher stimulation frequencies (400 sec^-1 ^and 600 sec^-1^), at which the CNCV values of both groups of nerves decreased by much larger percentages, with no difference between them. The Wedensky ratios (see Materials and Methods) and large (**L**) and small (**S**) amplitude decreases were analyzed at different stimulation frequencies to monitor the frequency-dependent conduction failure. At 100 sec^-1 ^and 600 sec^-1 ^stimulation, no significant difference between the Wedensky ratios for the *Hexb*+/- and *Hexb*-/- mice was detected. In addition, the data show that the **L **and **S **signals dispersed at similar rates in the *Hexb*+/- and *Hexb*-/- nerves at 400 sec^-1 ^and 600 sec^-1 ^stimulation. However, at 400 sec^-1^, the Wedensky ratio was significantly higher for the *Hexb*-/- nerves than the *Hexb*+/- nerves (p < 0.05, two-tailed, unpaired *t*-test). To analyze the effects of stimulation frequency on the Wedensky ratio, the two values were plotted against one another (Figure 1). The slopes of the linear regressions for the *Hexb*-/- and *Hexb*+/- data did not differ significantly within 95% confidence limits.

**Table 1:** Sciatic Nerve Conduction Studies in *Hexb+/- *and *Hexb-/- *Mice

	*Hexb+/-*		*Hexb-/-*
CNCV (m/s)^a^	23.6 ± 0.6 (8)		25.1 ± 0.9 (9)

	100 sec^-1^	400 sec^-1^	600 sec^-1^

Percent ΔCNCV^a^			
*Hexb+/-*	2% ± 1 (7)	20% ± 4 (**L**) (7)	18% ± 4 (**L**) (6)
		21% ± 4 (**S**) (7)	19% ± 3 (**S**) (6)
*Hexb-/-*	5% ± 1 (9)*	20% ± 2 (**L**) (9)	22% ± 3 (**L**) (8)
		22% ± 2 (**S**) (9)	22% ± 4 (**S**) (8)
Wedensky Ratio^a^			
*Hexb+/-*	0.99 ± 0.00 (7)	0.67 ± 0.08 (7)	0.46 ± 0.09 (6)
*Hexb-/-*	0.99 ± 0.00 (9)	0.84 ± 0.03 (9)*	0.58 ± 0.08 (8)
Amplitude Decrease Ratio^a^			
*Hexb+/-*	-	0.54 ± 0.06 (**L**) (7)	0.41 ± 0.06 (**L**) (6)
		0.37 ± 0.06 (**S**) (7)	0.17 ± 0.02 (**S**) (6)
*Hexb-/-*	-	0.50 ± 0.03 (**L**) (9)	0.39 ± 0.03 (**L**) (8)
		0.42 ± 0.03 (**S**) (9)	0.22 ± 0.04 (**S**) (8)

CNCV, compound nerve conduction velocity; Percent ΔCNCV, Wedensky Ratio, Amplitude Decrease Ratio, see Materials and Methods; **L**, **S**, see Figure 1

^a ^Values represent the mean ± SEM (n)

* p < 0.05 (two-tailed, unpaired *t*-test), *Hexb+/- *vs. *Hexb-/-*

### CNS myelin was hypomyelinated, PNS myelin was normal

**Figure 2:**
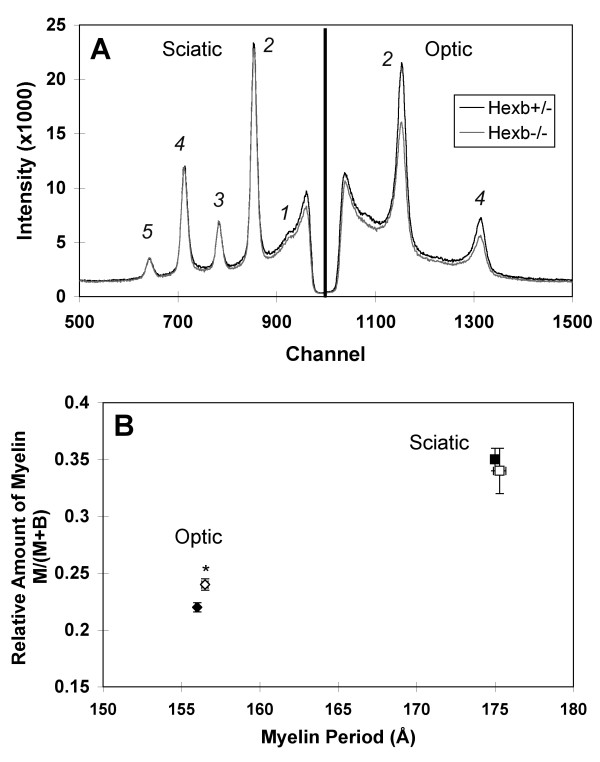
*Diffraction from Optic and Sciatic Nerves in Hexb+/- and Hexb-/- Mice*. **(A) **Representative examples of data for sciatic (left) and optic (right) nerves from *Hexb*+/- (black) and *Hexb*-/- (grey) mice. Whereas indistinguishable patterns were obtained for sciatic nerve samples from both groups, optic nerves from *Hexb-/- *mice showed weaker myelin scatter compared to those from *Hexb+/- *mice. The Bragg orders for the x-ray peaks are indicated as 1–5. **(B) **The fraction of total x-ray scatter (*M+B*) that is accounted for by compact myelin (*M*) (i.e., *M*/(*M+B*)), was plotted against the myelin period (*d*) [16]. For optic nerve myelin, the *Hexb*+/- (○) and *Hexb*-/- (●) mice have similar periods; however, the *Hexb*-/- mice have less relative myelin in the CNS when compared to the *Hexb*+/- mice (n = 3–4 per group, p < 0.05; two-tailed, unpaired *t*-test). For sciatic nerve, the *Hexb*+/- (□) and *Hexb*-/- (■) mice have similar periods and relative amounts of compact myelin (n = 8 per group). Thus, x-ray diffraction revealed no myelin abnormalities in the PNS and less relative amounts of compact myelin in the CNS of the *Hexb*-/- mice.

XRD analysis (Figure 2) revealed that the myelin period of optic nerves (CNS) for the *Hexb*+/- and *Hexb*-/- mice were 156.2 Å ± 0.2 (n = 3) and 156.0 Å ± 0.1 (n = 4), respectively (mean ± SEM). Myelin period of sciatic nerves (PNS) for the *Hexb*+/- and *Hexb*-/- mice were 175.3 Å ± 0.4 (n = 8) and 175.0 Å ± 0.3 (n = 8), respectively. Based on the relative strengths of the diffraction patterns [16,17], the relative amounts of myelin in the optic nerves of the *Hexb*+/- and *Hexb*-/- mice were 0.24 ± 0.01 (n = 3) and 0.22 ± <0.00 (n = 4), respectively. This suggests slightly less relative amounts of myelin in the optic nerve of the *Hexb*-/- mice (p < 0.02; two-tailed, unpaired *t*-test). By contrast, the relative amounts of myelin in the sciatic nerves of the *Hexb*+/- and *Hexb*-/- mice were indistinguishable (0.34 ± 0.02 (n = 8) and 0.35 ± 0.03 (n = 8), respectively).

**Figure 3:**
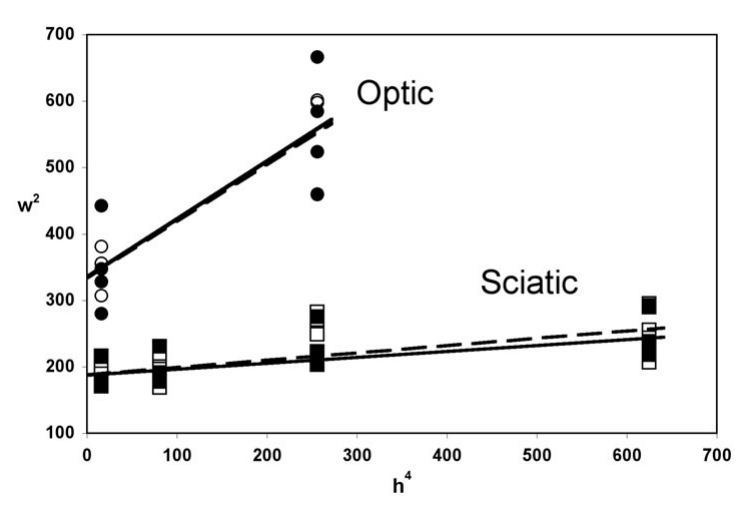
*Myelin Membrane Packing in Optic and Sciatic Nerves from Hexb+/- and Hexb-/- Mice*. The integral widths *w*^2 ^are plotted as a function of *h*^4 ^to determine the relative amount of myelin packing disorder according to the theory of paracrystalline diffraction [18]. The projected intercept on the ordinate axis is inversely related to the number of repeating units *N *(the coherent domain size), and the slope is proportional to the fluctuation in period, Δ (lattice or stacking disorder). There were no differences within 95% confidence levels between the *Hexb+/- *(open symbols, dashed line) and *Hexb-/- *(filled symbols, solid line) slopes of the optic (circles) or sciatic (squares) nerves (n = 3–8) indicating no change in the membrane packing of the internodal compact myelin for the sciatic nerves (PNS) and for the optic nerves (CNS).

The widths (*w*) of the x-ray peaks provide information about the relative number of myelin layers in a diffracting region of the sheath and the regularity of the membrane packing [16]. When the squares of the integral widths (*w*^2^) are plotted against the fourth power of the Bragg order (*h*^4^), the *y*-intercept of the trend-line is inversely proportional to the number of the repeating units (i.e., myelin membrane pairs) and the slope is proportional to the membrane packing disorder [18] (Figure 3). In the CNS, the slope for the *Hexb*+/- samples was 0.85 ± 0.12 with a *y*-intercept of 334 ± 20, and for the *Hexb*-/- samples the slope was 0.87 ± 0.15 with a *y*-intercept of 335 ± 35. In the PNS, the slope for the *Hexb*+/- samples was 0.11 ± 0.01 with a *y*-intercept of 188 ± 6, and for the *Hexb*-/- samples was 0.09 ± 0.01 with a *y*-intercept of 187 ± 8. These differences in the myelin packing and thickness between the *Hexb*+/- and *Hexb*-/- mice were not statistically significant. In accordance with recently published data [16], the steeper slope and higher y-intercepts for the optic nerve indicate that its myelin sheaths are thinner and have more packing disorder than myelin in the sciatic nerves.

### GM2 present in PNS

**Figure 4:**
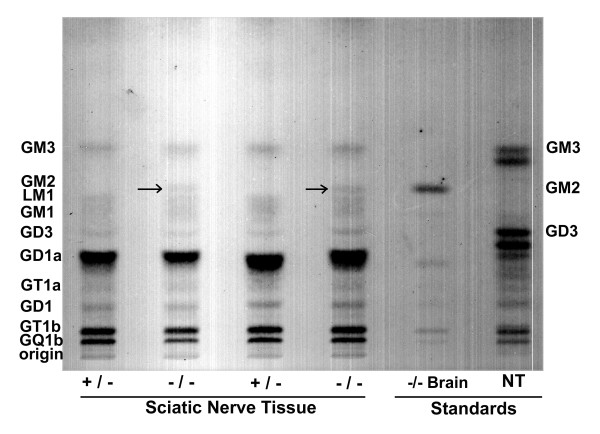
*HPTLC of Ganglioside Distribution in Hexb+/- and Hexb-/- Mice*. HPTLC of two *Hexb+/- *and two *Hexb-/- *samples show the ganglioside distribution of sciatic nerve tissue. For each sample, gangliosides having approximately 1.3 μg of sialic acid were spotted on the HPTLC plates. The plates were developed by a single ascending run with chloroform:methanol:*d*H_2_O (55:45:10, v:v) containing 0.02% CaCl_2_·2H_2_O. GM2 is present in the *Hexb-/- *lanes (arrows) and undetectable in the *Hexb+/- *lanes. The identity of the GM2 band was confirmed using an external standard (*Hexb-/- *brain tissue, neural tube).

The total ganglioside content of the sciatic nerves in the *Hexb*+/- and *Hexb*-/- mice was analyzed and the results are expressed as μg sialic acid/100 mg dry weight (mean ± SEM) (Table 2). No significant difference in total gangliosides was detected between the *Hexb*+/- and *Hexb*-/- samples. The ganglioside distribution of the sciatic nerves was determined from densitometric scanning of the HPTLC plate (Figure 4). 

The most noticeable difference was the presence of GM2 in *Hexb*-/- compared to *Hexb*+/- mice (Table 2). The *Hexb*-/- samples contained 1.0 and 0.9 μg sialic acid/100 mg dry weight of GM2 and neither *Hexb*+/- sample had any detectable levels of GM2. The presence of GM2 is apparent in the *Hexb*-/- sample lanes (Figure 4). No statistically significant differences were detected among the distribution of the other gangliosides, neutral lipids, and acidic lipids (Table 2).

**Table 2:** Lipid Distribution of Sciatic Nerve in *Hexb *Mice^a^

Lipids	*Hexb*+/-	*Hexb*-/-
Total Gangliosides	44.2 ± 1.2	39.7 ± 3.8
	(n^b ^= 5)	(n = 5)
		
Individual Gangliosides^c ^(n = 2)		
GM3	2.0, 2.5	2.8, 1.9
GM2	n.d.^d^	1.1, 1.0
LM1	1.5, 3.5	1.4, 0.8
GM1	0.7, 1.4	1.8, 1.0
GD3	0.6,0.6	0.6, 0.8
GD1a	23.1, 23.9	23.8, 19.0
GT1a	0.3, 0.3	1.0, 0.4
GD1b	1.4, 1.8	1.6, 1.5
GT1b	8.7, 7.7	8.6, 7.0
GQ1b	6.5, 5.1	5.1, 4.5
		
Neutral		
Triglycerides	38.2 ± 2.4	43.6 ± 5.0
Cholesterol	9.9 ± 1.2	11.4 ± 1.5
Cerebrosides	6.6 ± 0.5	6.7 ± 0.4
Phosphatidylethanolamine	5.4 ± 0.4	6.0 ± 0.4
Phosphatidylcholine	4.3 ± 0.3	3.8 ± 0.2
Sphingomyelin	2.6 ± 0.3	2.4 ± 0.4
	(n = 6)	(n = 6)
		
Acidic		
Sulfatides	1.4 ± 0.1	1.4 ± 0.1
Phosphatidylserine	3.2 ± 0.3	3.1 ± 0.1
Phosphatidylinositol	0.4 ± 0.0	0.4 ± 0.0
	(n = 5)	(n = 6)

^a ^Values are expressed as mean ± SEM in mg/100 mg dry weight (neutral, acidic) or μg sialic acid/100 mg dry weight (ganglioside).

^b ^n, the number of independent samples analyzed. (6–8 sciatic nerves were pooled per independent sample)

^c ^due to small amounts of gangliosides present in tissue, only two samples were obtained for analysis

^d ^n.d., not detected

## Discussion

Brain dysmyelinogenesis is suspected as a secondary symptom of GM2 gangliosidoses [6,19-21]. Supporting this hypothesis, the present XRD results indicated hypomyelination in the amount of compact myelin in the optic nerve of *Hexb*-/- mice. According to these results, future lipid analysis of myelin isolated from optic nerves from *Hexb*-/- mice would be expected to show a slight reduction in cerebrosides and sulfatides, myelin markers. In response to these XRD results and the growing literature supporting myelin abnormalities in the CNS, the present study examined a number of PNS characteristics that would be affected if abnormal PNS myelin is present in the *Hexb*-/- mice.

The present electrophysiological studies indicated only slight variations in frequency-dependent conduction failure of excised sciatic nerve tissue between the *Hexb*+/- and *Hexb*-/- mice, and these changes were not observed consistently under the different stimulation conditions. In addition, there was no significant difference in the CNCV values. This is consistent with past case studies reporting normal motor conduction velocities in patients with chronic GM2 gangliosidosis [13] and with the results of the present study obtained from the PNS using XRD. These findings suggest that the structure and function of the nodal and paranodal regions are normal.

We used XRD here as a sensitive and quantitative probe of the relative amount of myelin and its periodicity in a large volume of unfixed tissue (i.e., whole sciatic and optic nerves) rather than from just a thin-section, as for electron microscopy. Previous measurements demonstrate the consistency of XRD findings with those from microscopy [16,17,22]. No significant differences between the *Hexb*+/- and *Hexb*-/- mice were found for the breadths of the x-ray reflections, which informs about average myelin thickness and membrane packing disorder. Together with the decrease of the relative amount of compact myelin detected in the optic nerve, these results suggest that the axon fiber density (number of axon fibers per cross-sectional area) in the optic nerves of the *Hexb*-/- mice may be less than in the *Hexb*+/- mice. A decrease in the axon fiber density in the *Hexb*-/- mice may be due to the neurodegeneration observed at late stages of disease progression in mouse models of SD [23,24]. Electron microscopy of optic nerve cross-sections would be required to test this hypothesis. Unlike the CNS findings, no reduction of compact myelin was detected in the PNS. In accordance with this finding, no change in the amount of cerebrosides or sulfatides was detected in the PNS tissue. Past studies have shown that LM1 is found to be mainly in rat PNS nerve myelin and that it deposits like cerebrosides and sulfatides. Therefore, relative amounts of LM1 could possibly be used as a marker for the amount of myelin in the PNS tissue if the ganglioside distribution in mouse PNS tissue is similar to that in rat PNS tissue [25]. In the future, lipid analysis of LM1 in myelin isolated from sciatic nerve samples could provide further verification of the present XRD results. Whether or not myelination was delayed in the CNS, as previously suggested [21], or in the PNS cannot be resolved from the present experiments. XRD analysis would be required at various age points during the progression of the disease to detect delayed myelination.

Our XRD findings indicating no decrease in the amount of compact myelin in the PNS seem inconsistent with the case study of an adult with GM2 gangliosidosis in which nerve biopsy of the peroneal nerve showed severe loss of myelinated fibers, especially those with the largest diameter [13]. One might expect that this would have a significant impact on the relative amount of compact myelin detected by XRD if similar loss of myelinated fibers in the PNS was present in the *Hexb*-/- mice. The discrepancy may be explained by the phenotypic differences between the two systems. The case study is an adult-onset variation of GM2 gangliosidosis, whereas the *Hexb*-/- mice resemble the infantile variant most closely [9]. Regarding electrophysiology, our recording set-up may not have been able to detect the behavior of the largest diameter myelinated fibers (fastest conducting fibers). We measured peak amplitudes and latencies that are not representative of the fastest fibers. Analysis of the behavior of these fibers was hindered by the overlap with the falling phase of the stimulus artifact. In future experiments, electron microscopy on cross-sectioned sciatic nerve could elucidate the relative ratios of large and small diameter nerve fibers in the sciatic nerves of the *Hexb*-/- mice.

Lipid analysis indicated no significant change in the *total *ganglioside content of the sciatic nerve tissue in the *Hexb*-/- mice; however, GM2 was increased. Previously, GM2 accumulation has been reported in the anterior horn motor neurons and Schwann cells in the dorsal root ganglion [9,14,15], regions that were not isolated with the peripheral tissue samples examined here. Therefore, the slight GM2 elevation we observed suggests GM2 storage throughout the PNS, and perhaps localized to the ensheathing Schwann cells. Lipid analysis of myelin isolated from sciatic nerve of the *Hexb*-/- mice would be necessary to confirm this. This accumulation may be partly responsible for the phenotypic symptoms observed in the *Hexb-/- *mice. Lipid analysis also revealed that the ganglioside composition in the mouse PNS is very different from the previously reported ganglioside composition in the mouse brain of the *Hexb*+/- mice [6]. GM3 was found in small amounts and GD1a was the major ganglioside in the *Hexb*-/- and *Hexb*+/- samples. These results also differ from previously reported ganglioside distribution for mouse sciatic nerve [25]. A difference in the mouse strain and age may account for the discrepancies.

## Conclusion

In summary, these experiments offer evidence for dysmyelination in the CNS in SD models. PNS findings suggest that peripheral symptoms observed in SD models stem from abnormalities in the CNS. Further studies will be necessary to elucidate the extent to which the PNS is involved in the pathology of SD and to determine the usefulness of targeting this system during treatment design.

## Methods

### Transgenic mice

Sandhoff mice (*Hexb-/-*), derived by homologous recombination and embryonic stem cell technology [26], were obtained from Dr. Richard Proia (National Institutes of Health, Bethesda, MD, USA). The heterozygous (*Hexb+/-*) and knockout (*Hexb-/-*) mice that were used during these experiments were bred at the Boston College Animal Facility by crossing *Hexb+/- *females with *Hexb-/- *males. *Hexb+/- *animals exibit identical lipid profiles as *Hexb+/+ *animals, show no phenotype, and live a normal mouse life span [9]. To ensure the genotype of the mice, the hexosaminidase specific activity was measured from tail tissue using a modified Galjaard procedure [27,28]. All mice were kept in individual plastic cages with filter tops containing Sani-Chip bedding and cotton nesting pads. The room was kept at 22°C on a 12 h light and 12 h dark cycle and were fed Prolab RMH 3000 chow (LabDiet, Richmond, IN, USA). All animal experiments were carried out in accordance with the Boston College Institutional Animal Care and Use Guidelines.

### Electrophysiology

Mice were sacrificed around 4 months of age (120 – 142 days) by cervical dislocation and decapitation. Sciatic nerves were immediately dissected from the ankle to the spinal column (1.6 – 2.5 cm) and placed in Locke solution (154 mM NaCl, 5.6 mM KCl, 2.2 mM CaCl_2_, 5 mM dextrose, 2 mM HEPES, pH 7.2) at room temperature. The nerve chamber contained circulating Locke solution that was equilibrated to 28°C using a Peltier device. This temperature was chosen instead of body temperature in order to slow conduction and thereby maximize separation between the stimulus artifact and the CAP. In addition, 28°C is a temperature where metabolism is sufficient to maintain ion gradients for several hours. At higher temperatures, the stimulus artifact and action potential overlapped owing to the small length of the nerve. After immersing the nerve in the 28°C solution, the proximal end was laid across a pair of stimulating Ag/AgCl electrodes above the solution. The distal end was then drawn into a suction electrode containing a Ag/AgCl wire and also lifted above the solution. To minimize the size of the stimulus artifact and maximize the size of the CAP signal, the diameter of the suction electrode matched the diameter of the nerve where the two made contact. Between the stimulating and recording electrodes, the nerve remained immersed in the circulating, 28°C Locke solution. Cathodal stimulation was employed for all of the experiments. Stimulus duration was set to 0.035 ms and the supramaximal stimulus (Grass Instruments, Quincy, MA, USA) was determined by monitoring the height of the CAP on an oscilloscope (Tektronix, Beaverton, OR, USA). The stimulus voltage was increased until the height of the CAP no longer increased. Then, raising the stimulus by 25%, the supramaximal stimulus was obtained. Throughout the experiment, one minute of resting activity was maintained between each recording. Recordings at high frequency stimulation were taken for 1 second. Waveforms were captured using model 1401 A/D converter (Cambridge Electronic Design, Cambridge, UK). Data were analyzed off-line using *Spike 2 *software (Cambridge Electronic Design, Cambridge, UK). Frequency-dependent procedures were organized as follows: 3 single CAPs, 3 at 100 sec^-1^, 3 single CAPs, 3 at 400 sec^-1^, 3 at 600 sec^-1^, and 3 single CAPs.

Compound nerve conduction velocity values were determined for each nerve by dividing the length of the nerve by the latency between the stimulus and the highest point of the earliest CAP peak (T_l_; see Figure 1), corresponding to the maximum sum of the APs of the fastest conducting axons. The values from the ~18 recordings for each nerve were averaged to yield the representative CNCV value for that nerve. The CNCV values are presented as mean ± standard error for the *Hexb*-/- and *Hexb*+/- mice. A two-tailed, unpaired *t*-test was applied to determine any significant differences between the two groups. The reversible depression of the CAP signal that is observed during a period of high frequency stimulation is due to both the differential slowing of conduction (dispersion) and to the alternating conduction failure among the myelinated axons within a nerve. This latter phenomenon, known as Wedensky inhibition (in which the CAP amplitudes alternated between small and large), was quantitated by comparing the amplitude above the pre-stimulus baseline of the first CAP signal in a train of impulses to those of the last 6 CAP signals in the train. At 400 sec^-1 ^and 600 sec^-1 ^stimulation, when such Wedensky inhibition was observed, the small (**S**) and large (**L**) amplitudes at the end of the train were analyzed separately to monitor the behavior of the conduction failure (Figure 1). Ratios for the **S **to the first CAP amplitude, **L **to the first CAP amplitude, and **S **to **L **("Wedensky ratio") were calculated. Two-tailed, unpaired *t*-tests were employed to determine the p values between the *Hexb*-/- and the *Hexb*+/- mice for these different conduction parameters. For all results, sample values that were greater or less than the mean value by 6 standard errors were not included in the analysis.

### X-ray diffraction and myelin structure analysis

Nerve tissue samples were prepared for XRD as described [16]. Mice were sacrificed around four months of age by cervical dislocation and the sciatic and optic nerves were immediately dissected by tying them off at both ends with silk suture. The nerves were continually rinsed with physiological saline (154 mM NaCl, 5 mM Tris buffer, pH 7.4) during the dissection. The nerves were slightly extended in 0.7-mm (sciatic nerves) or 0.5-mm (optic nerves) quartz capillary tubes (Charles Supper Co., Natick, MA, USA) containing saline. The capillaries were then sealed at both ends with wax.

XRD experiments utilized nickel-filtered, single-mirror-focused CuKα radiation from a fine-line source on a 3.0 kW Rigaku x-ray generator (Rigaku/MSC Inc., The Woodlands, TX, USA) operated at 40 kV by 14 mA. In accordance with our established protocol [16], XRD patterns for each sample were recorded for 1 h using a linear, position-sensitive detector (Molecular Metrology, Inc., Northampton, MA, USA). The diffracted intensity was then input into Excel, and the corresponding intensities from each side of the beam stop were averaged to obtain a more accurate measurement of the myelin periodicity, which is calculated from the positions of the peaks. The intensity data was subsequently input into PeakFit (Jandel Scientific, Inc.) and the background was subtracted. The intensity of the resulting peaks was integrated to obtain integral areas *I*(*h*) and integral widths *w*(*h*) for each reflection of order *h*. To determine the relative amounts of myelin packing disorder, the integral widths *w*^2 ^were plotted as a function of *h*^4^, in which the intercept on the ordinate axis is inversely related to the number of repeating units *N *(the coherent domain size), and the slope is proportional to the fluctuation in period Δ (lattice or stacking disorder) [18]. Lastly, the relative amount of compact myelin in the whole nerve was estimated by summing the integrated intensity for myelin (*M*) after background (*B*) subtraction (excluding the small-angle region around the beam stop and the wide-angle region of the pattern). A scatterplot of the fraction of total, integrated intensity that is a result of myelin (*M*/(*M+B*)) versus myelin period (*d*) [16] was used to determine whether there are differences in the myelin period and/or the relative amount of compact myelin between the two groups of transgenic mice.

### Lipid isolation, purification, and quantification

Total lipids were isolated from mouse brain standards and sciatic peripheral nerve tissue for analysis using established protocols [29]. To prepare the samples, 40 *Hexb*+/- and 38 *Hexb*-/- mice were sacrificed around 4 months of age. Due to insufficient amount of tissue, lipid analysis of optic nerve was not conducted. Each sciatic nerve sample contained nerves from 6–8 mice. After storage at -80°C, the samples were lyophilized overnight and the lipids were prepared as previously described [29]. Briefly, total lipids were extracted using chloroform:methanol (1:1, v:v) and *d*H_2_O, then resuspended in chloroform:methanol:water (30:60:8, v:v), and applied over a DEAE-Sephadex A-25 Column (Pharmacia Biotech, Uppsala, Sweden). The eluant was collected as the F1 fraction, which contains the neutral lipids cholesterol, phosphatidylcholine, phosphatidylethanolamine, plasmalogens, ceramide, sphingomyelin, and cerebrosides. The F2 fraction, which contains the gangliosides and the acidic lipids, was then eluted from the column with chloroform:methanol:0.8 M sodium acetate (30:60:8, v:v). To further purify the F2 fraction, the samples were subjected to the Folch procedure, which separated the gangliosides and salts (upper aqueous phase) from the acidic lipids (lower organic phase) [30,31]. The ganglioside fraction was then further purified by base treatment with sodium hydroxide followed by desalting using a C18 reverse-phase Bond Elute column (Varian, Harbor City, CA). Total gangliosides were quantified using the resorcinol assay previously described [29]. Svennerholm nomenclature for gangliosides is used [32].

All lipids were analyzed qualitatively by high-performance thin-layer chromatography (HPTLC) using previously described methods [29]. Briefly, for gangliosides, 1.5 μg sialic acid was spotted per lane. Due to the small amount of gangliosides present in the sciatic nerve, ganglioside samples were pooled to obtain an N of 2. The plates were developed by a single ascending run with chloroform:methanol:*d*H_2_O (55:45:10, v:v) containing 0.02% CaCl_2_·2H_2_O. Gangliosides were visualized using a resorcinol-HCl reagent and heating at 105°C for 30 min. For acidic lipids, 100–200 μg dry weight of each sample was spotted, and for neutral lipids, 35–70 μg dry weight of each sample was spotted. An internal standard (oleoyl alcohol) was added to both the lipid standards and to the samples as previously described [33]. The neutral and acidic lipid plates were developed with chloroform:methanol:acetic acid:formic acid:water (35:15:6:2:1, v:v) to a height of 4.5 cm or 6.0 cm, respectively, and then developed completely with hexanes:diisopropyl ether:acetic acid (65:35:2, v:v). The plates were subsequently charred with 3% cupric acetate in 8% phosphoric acid solution followed by heating at 160°C for 7 min for visualization.

To quantify the ganglioside results, the percentage distribution and density of the individual bands were determined by scanning the plates on a Personal Densitometer SI with ImageQuant software (Molecular Dynamics, Sunnyvale, CA, USA). The total ganglioside distribution was normalized to 100%, and the percentage distribution values were used to calculate sialic acid concentration (μg of sialic acid per 100 mg dry weight) of individual gangliosides [34]. The results for both neutral and acidic lipids were quantified using the same technique described for the gangliosides except the density values for the lipids were fit to a standard curve of the respective lipid and used to calculate individual concentrations (mg per 100 mg dry weight).

## Abbreviations

SD = Sandhoff disease

PNS = peripheral nervous system

CNS = central nervous system

GSL = glycosphingolipid

XRD = low-angle x-ray diffraction

CNCV = compound nerve conduction velocity

*w *= integral width

*h *= Bragg order

*M *= integrated intensity for myelin

*B *= background intensity

*d *= myelin period

## Competing interests

The author(s) declare that they have no competing interests.

## Authors' contributions

MAM conceived the study, conducted x-ray experiments, electrophysiological experiments, lipid analysis, and drafted the manuscript. RCB ran parallel lipid analysis (data included). RLA established x-ray diffraction protocol. TNS participated in the design of the study. GRS developed the electrophysiology experiment and analysis protocol. DAK established the analysis protocol for x-ray diffraction, participated in the design of the study, and helped draft the manuscript. All authors read and approved the final manuscript.
